# Multiscale Kernel-Based Residual CNN for Estimation of Inter-Turn Short Circuit Fault in PMSM

**DOI:** 10.3390/s22186870

**Published:** 2022-09-11

**Authors:** Qiang Song, Mingsheng Wang, Wuxuan Lai, Sifang Zhao

**Affiliations:** National Engineering Laboratory for Electric Vehicles, Beijing Institute of Technology (BIT), Beijing 100081, China

**Keywords:** multiscale architecture, fault diagnosis, inter-turn short circuit (ITSC) fault, permanent magnet synchronous motors (PMSM), dilated convolutional neural networks (CNN)

## Abstract

The diagnosis of an inter-turn short circuit (ITSC) fault at its early stage is very important in permanent magnet synchronous motors as these faults can lead to disastrous results. In this paper, a multiscale kernel-based residual convolutional neural network (CNN) algorithm is proposed for the diagnosis of ITSC faults. The contributions are majorly located on two sides. Firstly, a residual learning connection is embedded into a dilated CNN to overcome the defects of the conventional convolution and the degradation problem of a deep network. Secondly, a multiscale kernel algorithm is added to a residual dilated CNN architecture to extract high-dimension features from the collected current signals under complex operating conditions and electromagnetic interference. A motor fault experiment with both constant operating conditions and dynamics was conducted by setting the fault severity of the ITSC fault to 17 levels. Comparison with five other algorithms demonstrated the effectiveness of the proposed algorithm.

## 1. Introduction

Owing to the advantages of high efficiency, high power density, and excellent torque control performance, permanent magnet synchronous motors (PMSMs) have been widely applied in various applications, such as domestic appliances, wind power generators, and electric vehicles [1]. With the diversity of applications, the reliability of the PMSM has gradually attracted more attention. The motor faults would lead to unexpected shutdown or even catastrophic consequences, especially in systems with high-security requirements [2]. Thus, to avoid disastrous results, the fault diagnosis of PMSM is crucial for system safety.

As one of the most common faults in PMSM, the stator winding inter-turn short circuit (ITSC) fault is usually hard to identify [3]. Moreover, without timely and appropriate management, the PMSM runs into a severe short circuit fault or even an open circuit fault [4]. The ITSC fault is formed by winding insulation failure, which is usually caused by overcurrent, thermal stress, mechanical stress, and aging [5]. When an ITSC fault happens, the shorted point forms an extra circuit connection, which is parallel with the fault-winding phase and coupled with other windings and rotor magnets, through flux linkages [6]. Then, an overcurrent is generated in the fault winding, causing a large amount of additional heat generated by ohmic losses, which can further intimidate the adjacent wires or even melt them down. As a consequence of the above description, in some cases, even a slight ITSC fault can quickly expand to neighboring wires, so that a slight fault easily develops into a critical one [7]. Therefore, it is very important to detect and manage an ITSC fault at its early stage.

Traditional machine learning fault diagnosis algorithms, such as artificial neural networks (ANN) and support vector machines (SVM), are usually used in conjunction with characteristic acquisition methods, as they commonly have difficulties in extracting complicated features [8,9]. Characteristic acquisition methods can convert the collected original signals into low dimensional characteristic vectors, which are comparatively constant with regard to conversions and deformations, making them easy to match. Most conventionally used characteristic acquisition methods, such as fast Fourier Transform (FFT), short-time Fourier Transform (STFT), wavelet transform (WT), Hilbert-Huang transform (HHT), empirical mode decomposition (EMD), and Wigner-Ville distributions, etc., are all widely adopted in motor fault diagnosis [10,11,12,13]. The quality of the acquired characteristics from the signals to be detected has a great influence on the performance of traditional machine learning algorithms.

Motors are becoming increasingly complex and diverse, which makes the selection of fault features for each type of motor particularly time-consuming. Besides, the configuration of the features acquisition algorithm requires relevant prior knowledge, which is highly targeted to different tasks, so it also consumes a lot of time [5]. On the other hand, with the development of sensor technology, the acquisition of data is becoming easier and easier. Therefore, traditional fault diagnosis methods are being revisited from the perspective of big data [14]. Currently, the usage of deep learning algorithms has led to a range of breakthroughs in the research of motor fault diagnosis thanks to the fascinating peculiarity that they can acquire high-level and hierarchical representations from huge original data straight away [15]. Recurrent neural networks (RNN), deep belief networks (DBN), convolutional neural networks (CNN), and sparse autoencoders (SAE) are favorite deep learning algorithms adopted in different fault diagnosis studies [16]. In [17,18] a new deep SAE algorithm was proposed to help to enhance the performance of the deep network by studying the related structure and configurational connection information among different fault states. Lee et al. combined RNN with an attention mechanism to realize the fault degree estimation of an ITSC fault [19]. Liu et al. proposed an RNN-based autoencoder algorithm to do the bearing fault detection of the motor [15]. Shao et al. proposed an improved convolutional DBN algorithm embedded with a compressive sensing mechanism to implement single and compound faults diagnosis using vibration signals [20]. Ince et al. proposed a one-dimensional (1-D) CNN algorithm to realize online bearing fault diagnosis only using simple CNN configurations [21]; although the method can only diagnose whether there is a fault or not. Lee et al. adopted 1-D CNN to acquire fault characteristics from current signals of a PMSM, accomplishing fault diagnosis of the demagnetization and ITSC faults simultaneously. Some other fault diagnosis studies also benefit a lot from deep learning algorithms [22].

Although studies on fault diagnosis using deep learning algorithms have yielded significant results, there has been little research on early fault diagnosis, let alone the early fault diagnosis of the ITSC fault. Early fault diagnosis is rather important in ITSC faults as the fault overcurrent and overheating can bring about more serious problems. Few studies have focused on the severity estimation of the ITSC fault, which has great significance in guiding the safe operational field of the motor. In addition, the adopted current signals are normally long 1-D signals, which are greatly affected by electromagnetic interference and change with operating conditions. Thus, the realization of ITSC fault diagnosis requires extracting deeper and higher dimensional features from collected current signals, especially for those working under dynamic operating conditions [23,24]. Meanwhile, if deep networks are adopted to diagnose the ITSC fault, deep 1-D network architectures are required to extract features from the complex current signals. However, tests reveal that when deep networks start to converge, a difficulty is exposed because, as increase of network depth occurs, the performance of the network tends to saturate or even decline, which is different from overfitting [25]. On the other hand, generally, the input data of deep networks are processed on a specific scale, which is caused by the specific single-scale convolutional kernel, or constant pooling size, whereas motors are usually working under dynamic conditions, which makes collected signals time-varying [26]. What is more, applying a conventional CNN in time-series signal missions is also a challenge because they can only look back on history with linear size, which greatly limits the use of CNN for missions demanding longer histories [27]. Hence, although conventional CNN is a valuable tool for data analysis, it is less efficient regarding time-series signals, especially time-varying signals. Furthermore, the performance of deep networks depends enormously on the amount and distribution of the data set [28]. To enhance the performance of deep networks the data set should cover enough working area and fault conditions, particularly those under dynamic operating conditions, which has been less emphasized in much research. Finally, as the data set increases, the hyperparameter tuning of the deep network consumes lots of time, especially for people who are not familiar with hyperparameter tuning. Therefore, how to speed up the whole process of hyperparameter tuning is also an essential task to be resolved.

To address the aforementioned problems, a multiscale residual dilated CNN architecture is proposed in this article for the ITSC fault diagnosis of the PMSM. The key contributions of this article are concluded as below:

(1) A well-designed deep learning network, termed multiscale residual dilated CNN, is proposed for the early-stage severity estimate of ITSC in a PMSM. In multiscale residual dilated CNN, multiscale dilated CNN is used to capture features from huge raw current signals in various scales, which enhances the characterization ability of captured features and the robustness of the network, even under dynamic operating conditions. In addition, the dilated CNN can extend the receptive field of the deep network at an exponential level. Moreover, the residual connection architecture is adopted to solve the performance degradation problem, making rather deep networks possible.
(2) The Bayesian optimization algorithm is adopted to settle the hyperparameter tuning problem, which means the tuning process can be automated. What is more, the use of the Bayesian optimization algorithm suggests that comparisons between different methods becomes objective, as the tuning of hyperparameters is implemented automatically.
(3) The proposed algorithm has the advantage that it can extract features and diagnose faults from the huge raw current signals without the operating of signal processing, as it belongs to CNN. This also indicates that the relevant learning time of prior knowledge of fault characteristics can be saved.
(4) The proposed algorithm is validated by a motor fault experiment with both constant operating conditions and dynamic operating conditions. The result is compared with five other diagnosis algorithms to exhibit the advantages of the proposed algorithm.

The rest of this paper is arranged as follows. Section 2 derives an index to instruct the severity settings for ITSC faults. Section 3 explains the whole structure and components of the proposed algorithm. The experimental setup, severity setting of the ITSC fault, and data set description are described in Section 4. In Section 5, the proposed algorithm and five other deep learning algorithms are adopted to diagnose the same experimental test data set, which exhibits the effect and superiority of the proposed algorithm. Lastly, Section 6 concludes this paper and gives an outlook on the next steps.

## 2. ITSC Fault in PMSM

The detection of ITSC faults is very important as an overcurrent and overheating can lead to more serious problems. In previous studies, no index has been especially well-suited for guiding the early-stage fault severity setting of an ITSC fault. In this paper, an index is proposed for guiding the severity setting of the ITSC fault experiment.

**Figure 1:**
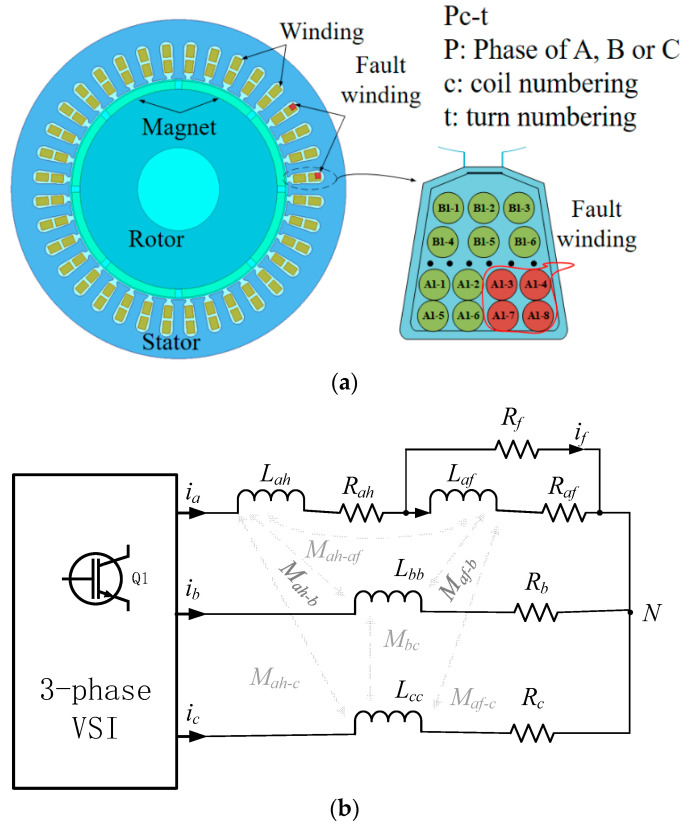
(**a**) Cross section of 8-pole-36-slot PMSM with an inter-turn short fault in coil a1. (**b**) Equivalent circuit model of inter-turn short fault in coil A1.

A cross-sectional view of a PMSM with an ITSC fault is shown in Figure 1a. The motor is a concentrated winding structure with 8 poles and 36 slots. Wherever the shorting point occurs in the coil, the wires in the corresponding slots are shorted accordingly, as shown by the red wires in Figure 1a. The Pc-t represents the unique numbering of every wire in one slot. Take A1-3, for example, it represents the third turn of wire in the first coil within phase A. Assuming the ITSC fault occurs in the first coil of phase A, the equivalent circuit model is shown in Figure 1b. When the fault occurs, an additional circuit that is in parallel with the fault windings of the same phase is formed. According to Figure 1b, the equivalent circuit model can be described as follows.

(1) $$\left[v_{abc,N}\right]=\left[R_{abc,f}\right]\left[i_{abc,f}\right]+\frac{d}{dt}(\left[L_{abc,f}\right]\left[i_{abc,f}\right]+\left[\lambda_{abc,f}\right])$$

where

$$\left[v_{abc,N}\right]={\left[\begin{array}{cccc} v_{a} & v_{b} & v_{c} & 0 \end{array}\right]}^{t}-{\left[\begin{array}{cccc} v_{N} & v_{N} & v_{N} & 0 \end{array}\right]}^{t}$$

$$\left[R_{abc,f}\right]=\left[\begin{array}{cccc} R_{a} & & & \mu R_{a}\\ & R_{b} & & \\ & & R_{c} & \\ \mu R_{a} & & & \mu R_{a}+R_{f} \end{array}\right]$$

$$\left[i_{abc,f}\right]={\left[\begin{array}{cccc} i_{a} & i_{b} & i_{c} & i_{f} \end{array}\right]}^{t}$$

$$\left[L_{abc,f}\right]=\left[\begin{array}{cccc} L_{aa} & M_{ab} & M_{ac} & \mu L_{aa}\\ M_{ab} & L_{bb} & M_{bc} & \mu M_{ab}\\ M_{ac} & M_{bc} & L_{cc} & \mu M_{ac}\\ \mu L_{aa} & \mu M_{ab} & \mu M_{ac} & \mu^{2}L_{aa}N_{c} \end{array}\right]$$

$$\left[\lambda_{abc,f}\right]={\left[\begin{array}{cccc} \lambda \cos\theta_{e} & \lambda \cos(\theta_{e}-\frac{2}{3}\pi ) & \lambda \cos(\theta_{e}+\frac{2}{3}\pi ) & \mu \lambda \cos\theta_{e} \end{array}\right]}^{t}$$

*v_a_*, *v_b_*, and *v_c_* denote the phase voltage. *v_N_* denotes the voltage of the neutral point. *R_a_*, *R_b_*, and *R_c_* denote the phase resistance. *L_aa_*, *L_bb_*, and *L_cc_* denote the inductance of phase A, phase B, and phase C respectively. *M_ab_*, *M_bc_*, and *M_ca_* denote the mutual inductance between phase A, phase B, and phase C. The value *μ* denotes the shorted turn ratio, which indicates the ratio of shorted turns *N_c_* to the total number of turns *N_p_* in one phase. *R_f_* denotes the contact resistance between the shorted turns, *i_a_*, *i_b_*, and *i_c_* denote the phase current, *i_f_* denotes the fault current in the shorted path, and *λ* denotes the amplitude of the permanent magnet flux linkage. It can be seen from the model that the severity of the ITSC fault is influenced by the fault resistance *R_f_* and the shorted turn ratio *μ*.

According to Kirchhoff’s current law, the relationship of the currents that flow through the neutral point of the winding can be expressed as:

(2) $$i_{a}+i_{b}+i_{c}=0$$

Combining (1) and (2), the fault current *i_f_* can be derived as:

(3) $$i_{f}=\frac{\mu (v_{a}-v_{N})+(\mu^{2}L_{aa}-\mu^{2}L_{aa}N_{c})\frac{di_{f}}{dt}}{\mu R_{a}+R_{f}-\mu^{2}R_{a}}$$

Define *d*_1_ = *μRa* + *R_f_* − *μ*^2^*R_a_*, *d*_2_ = *μ*^2^*L_aa_* − *μ*^2^*L_aa_N_c_*, then (3) can be rewritten as:

(4) $$\frac{di_{f}}{dt}=\frac{1}{d_{2}}(d_{1})i_{f}-\frac{1}{d_{2}}\mu (v_{a}-v_{N})$$

As the amplitude of *v_N_* is much smaller than *v_a_* in the early stage of an ITSC fault, then *v_a_* ≈ *v_a_* − *v_N_*. Assuming *v_a_* = *V_a_*sin(*ωt*), the solution of (4) can be described as:

(5) $$i_{f}(t)=e^{\frac{d_{1}\;t}{d_{2}}}\;\left(i_{f}(0)-\frac{\mu \;V_{a}\;\omega }{d_{2}\;\left(\omega^{2}\;+\frac{d_{1}{}^{2}}{d_{2}{}^{2}}\right)}\right)+\frac{\mu V_{a}\;\left(\;\omega \;\cos\left(\omega t\;\right)+\frac{d_{1}\;\sin\left(\;\omega t\;\right)}{d_{2}}\right)}{d_{2}\;\left(\omega^{2}\;+\frac{d_{1}{}^{2}}{d_{2}{}^{2}}\right)}$$

where *N_c_* > 1, *R_f_* ≥ 0, 0 ≤ *μ* ≤ 1, *d*_1_ ≥ 0, *d*_2_ < 0. In the early stage of the fault, |*d*_1_| >> |*d*_2_|, the ratio of *d*_1_ to *d*_2_ tends to infinity while the ratio of *d*_2_ to *d*_1_ tends to 0. Combined with the above analysis, meanwhile, substituting the expression of *d*_1_ and *d*_2_ into (5), the amplitude of *i_f_* can be rewritten as:

(6) $$I_{f}\approx \frac{\mu V_{a}}{\mu R_{a}+R_{f}-\mu^{2}R_{a}}$$

Moreover, the amplitude of the three-phase voltage is proportional to the speed of the motor [29]. Then the relationship among parameters *I_f_*, *R_f_*, *μ*, and the speed of the motor *ω_r_* can be expressed as:

(7) $$I_{f}\propto \frac{\mu \omega_{r}}{\mu R_{a}+R_{f}-\mu^{2}R_{a}}$$

where *R_a_* can be treated as a known parameter. It can be seen from (7) that the parameters *μ*, *R_f_* and *ω_r_* directly influence *I_f_*. Among the related parameters, only *ω_r_* does not affect the ITSC fault, If divided from (7), a new expression that can reveal the severity of the fault to some extent can be derived:

(8) $$FI=\frac{I_{f}}{\omega_{r}}\propto \frac{\mu }{\mu R_{a}+R_{f}-\mu^{2}R_{a}}$$

where *FI* represents the fault index of the ITSC fault. When the motor is healthy, the index is 0. In the early stage of the fault, the index is almost invariant to the motor speed, and increases as *R_f_* decreases or *μ* increases. Each degree of ITSC fault can be seen as a unique combination of *R_f_* and *μ*. Since it is difficult to measure *R_f_* and *μ* during the operation of a motor, the index is not suitable to directly do the severity estimate of an ITSC fault. However, it can be used as an indicator to guide the severity setting of an ITSC fault.

## 3. Proposed Algorithm

### 3.1. Dilated Convolutional Neural Networks

The dilated CNN is a variant of conventional CNN, which contains dilated convolutional layers, RELU layers, a Batch normalization layer, and a dropout layer.

As a variant of conventional CNN, the dilated CNN inherits the characteristics of local connectedness and weight sharing and the result of the loss function can be optimized by a backpropagation algorithm [30]. The purpose of a convolution operation is to extract diverse levels of hierarchical features from the input signals. The top layers can only extract shallow features. The deeper a convolutional layer is, the more complicated the features that can be extracted from the input signals. The CNN achieves sparse connectivity by connecting neurons of neighboring layers using a local connection mode and adopting kernels of different sizes [26]. Thus, not only does it efficiently represent the complicated relationship, but it can also reduce the potential of overfitting.

The use of dilated CNN can eliminate the need for pooling layers, which allows the deep network to enlarge the receptive field, while minimizing the loss of coverage or resolution, making possible a rather deep network architecture [14,27,31]. For a 1-D series input data *S* ∈ *R^n^* and the kernel *f*: {0, 1,…, *k* − 1} → *R*, the expression of the dilated convolution *F* on input data segment *x* can be defined as:

(9) $$F(x)=(S{*}_{d}f)(x)=\sum_{i=0}^{k-1}f(i)\cdot S_{x-d\cdot i}$$

where *d* stands for the dilation factor, *k* denotes the filter size, and *x*-*d·i* indicates the element of segment *x* traversing the *i*-th convolution operation. Thus, the dilated convolution represents running convolution operations on elements of the series data by kernels whose filters are separated by a step size of *d*. Conventional convolution is a special case where the dilation factor *d* = 1. As the depth of the network grows, the dilated factor increases accordingly, and the receptive field of the final output layer becomes wider.

The rest layer in CNN helps to enhance the performance of the deep networks. The normalization layer is used to get rid of possible gradient disappearance and explosion problems, and the method here adopted is Batch Normalization. The activation layer is used to extend the nonlinearity of a neuron. We adopted rectified linear unit (ReLU) as our activation function to speed up the training process. To address the overfitting problem of the networks, some proportion of neurons and their connections can be randomly dropped, namely the dropout layer.

### 3.2. Residual Learning Block

The object that is analyzed for the diagnosis of an ITSC fault is the three-phase current of the PMSM. Current signals are usually long 1-D signals, which are highly susceptible to changeable operating conditions and electromagnetic interference, making the motor fault diagnosis more difficult. If the CNN is adopted to do the severity estimate of the ITSC fault, deeper 1-D architecture CNN is required, because normally the deeper a CNN architecture is, the more complex the features it can extract. Whereas former experiments proved that the CNN had a degradation difficulty, namely with the increase of CNN depth, the performance of the network tended to saturate and even degrade. That is to say, the addition of network depth decreases its performance, and the phenomenon is different from overfitting.

The degradation difficulty indicates that it is hard to train a CNN well. Theoretically speaking, if an added CNN layer just repeats the features of former layers, rather than learning new features, namely, the identity map, the performance of the network should not decrease [32]. Inspired by this instinct, the residual learning block was employed in the proposed algorithm. For a residual learning block, if the input is defined as *x*, the learned feature is denoted as *F*(*x*), which should be added to the input of the residual learning block. The output of the residual learning block is expressed as:

(10) y=σ(x+F(x))

where *y* stands for the output of the residual learning block, and σ denotes the activation function of the residual learning block.

Assuming the result of a residual learning block is greater than 0, the performance of the CNN can be further enhanced by increasing the depth of layers. On the contrary, if the result of the residual learning block is 0, the newly added layer does not influence the performance of the residual learning block, which is called an identity map. Hence, a deeper CNN architecture can be formed by adopting residual learning blocks to prevent the degradation difficulty.

**Figure 2:**
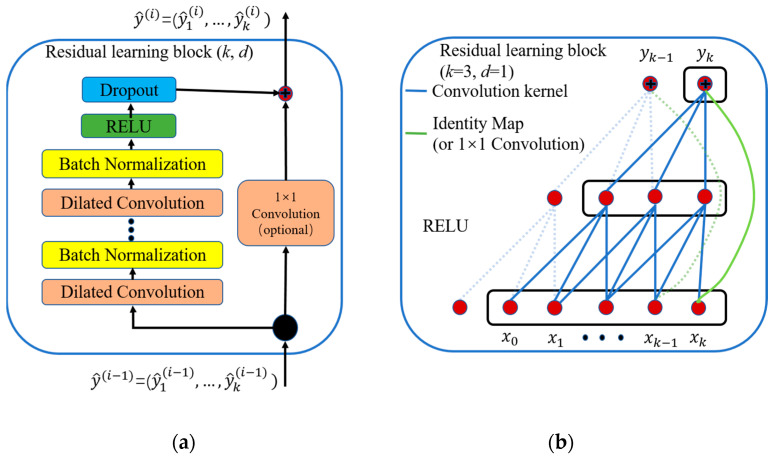
(**a**) Residual connection. A 1 × 1 convolution is added when the input and output of the residual connection have different dimensions. (**b**) An example of residual connection. The blue lines are convolution kernels in the residual connection, and the green lines are identity mapping of the residual connection.

The residual learning block is shown in Figure 2. As Figure 2a shows, the residual learning block is realized by stacking several layers of dilated convolution layer, Batch Normalization layer, RELU layer, and dropout layer. In a standard Residual Neural Network, the output of the block is added to the input without any transformation, (as shown in Figure 2b). Whereas, for the condition of 1-D CNN, an extra 1 × 1 convolution is used to avoid the situation of tensor disagreement between the input and output of the block.

### 3.3. Multiscale Kernel Dilated CNN

The essence of ITSC fault diagnosis is the use of time-series signals to achieve fault identification or regression. However, there are still some problems that need to be solved: firstly, the conventional convolution usually extracts features in the same scale size, namely single-scale. Whereas the signals of the motor are always changing with system structure, electromagnetic interference, and sampling rates, which indicates that a single-scale convolutional kernel size is not enough for extracting the signal features [33]. Secondly, the operating conditions of motors are always dynamic, even the setting of constant operating conditions is highly susceptible to changing loads, power supply stability, and environmental factors. Thus, the potential to evaluate signals over dynamic operating conditions should be taken into account if the fault diagnosis algorithm is intended to be adapted in more situations.

**Figure 3:**
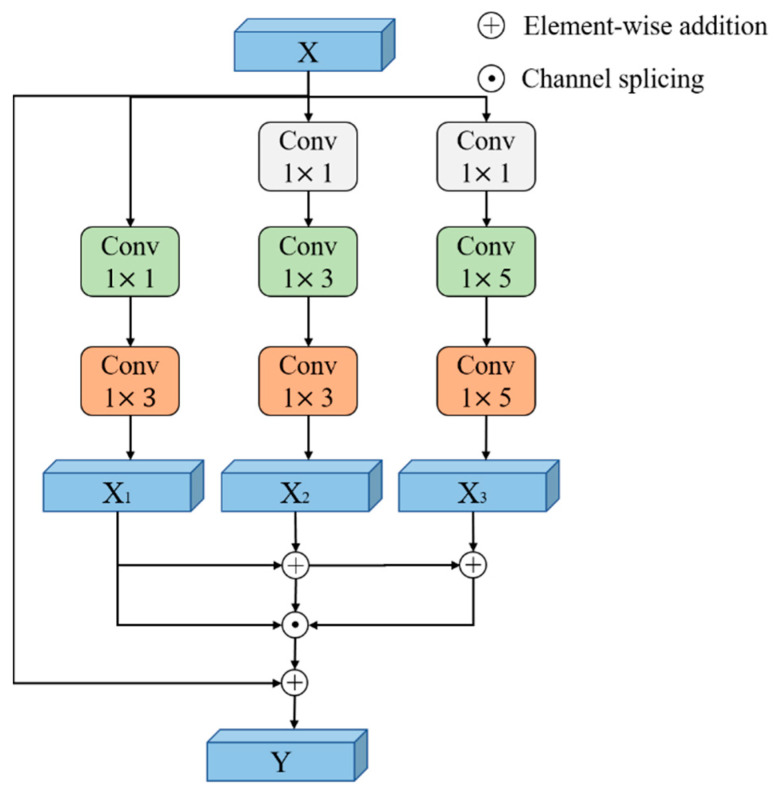
The architecture of a multiscale kernel-based dilated CNN.

To address these difficulties, a multiscale kernel-based dilated CNN architecture is proposed in this paper. In contrast to the multiscale of conventional CNN, which can only change the size of the extracted features by the size of the convolutional kernel, dilated CNN can also change the extracted features by changing the dilated factor *d* [34]. By influencing the receptive field of the network, the kernel size *f* and dilated factor *d* achieve extraction of different sized features. The formula for the receptive field is defined as:

(11) $$r_{f}={\left((d-1)(f-1)+f+2\right)}^{2}$$

where *r_f_* represents the size of the receptive field. As the dilated convolution does not sample the dilated parts, this results in the extracted information not being continuous. Hence, to extract the feature information of the dilated parts, a multiscale kernel-based dilated convolution architecture is proposed in this paper. As shown in Figure 3, the conventional convolution, with kernel sizes of 1 × 1, 1 × 3, and 1 × 5, is added before the dilated convolution of each branch is separately added. The red boxes of the figure show the features extracted by the dilated convolution, and the rest are the features extracted by conventional convolution. After adding the conventional convolution, the information of the dilated parts can be extracted by the conventional convolution, which can enhance the continuity of the information and obtain different sizes of receptive fields, realizing the extraction features at different scales. *X* is the input feature vector, and *Y* is the output feature vector. The purpose of conventional convolution with a kernel size of 1 × 1 is to decrease the number of channels, which can also reduce the amount of computation. The dilated convolution size of each branch is set to 1 × 3, and the dilated factor of each branch is set to *d* = 1, *d* = 3, and *d* = 5. The features of the three branches are spliced together after extracting different scale sizes of features by using different sizes of dilated factors. The expression of the features extracted by the three branches are:

(12) $$\begin{array}{l} X_{1}=X*C_{1\times 1}*D_{f=1}\\ X_{2}=X*C_{1\times 1}*C_{3\times 3}*D_{f=3}\\ X_{3}=X*C_{1\times 1}*C_{5\times 5}*D_{f=5} \end{array}$$

where *X*_1_, *X*_2_, and *X*_3_ are the features of each branch separately, the symbol * is the convolution operation, and *C*_1×1_, *C*_1×3_, and *C*_1×5_ represent the conventional convolution with kernel sizes of 1 × 1, 1 × 3, and 1 × 5 separately. *D_f_*_=1_, *D_f_*_=3_, and *D_f_*_=5_ represent the dilated convolution with a kernel size of 1 × 3 and dilated factors of 1, 3, and 5.

In this architecture, the way of feature fusion is layer-by-layer stacking. Thus, the output of the architecture is expressed as:

(13) $$Y=\{X_{1},X_{1}+X_{2},X_{1}+X_{2}+X_{3}\}$$

where the symbol {+} represents the element-wise addition operation, and {·} represents the splicing operation of different channels. The operation of adding up the features from dilated factor *d* = 1, to *d* = 3 and *d* = 5, layer-by-layer, makes the branch of *d* = 3 and *d* = 5 not only contain the feature with larger dilated factors but also contain the feature with small dilated factor, which enhances the network’s ability in extracting different scales of features.

### 3.4. Bayesian Optimization for Hyperparameter Tuning

The performance of the deep networks is highly dependent on an appropriate set of hyperparameters. However, there are interactions between different hyperparameters, and it is difficult to tune a set of appropriate hyperparameters without experience, and, even if you could, it takes a lot of time. Moreover, when compared with other deep learning algorithms, if the function of automatic hyperparameter tuning can be implemented, the introduction of subjective intent can be avoided, which makes the comparison of different algorithms more objective. Therefore, it is necessary to realize the automatic hyperparameter tuning by using the optimization algorithm that not only has the capability of global optimization but can also get good results in fewer iterations or taking less time.

The Bayesian optimization algorithm can realize highly efficient parameter optimization with a fast optimization rate and few iterations [32]. By iterating and evolving a global statistical model that has no clear target function, the Bayesian optimization algorithm achieves the estimation and evaluation of a task [35]. It mainly consists of two parts: one is the acquisition function and the other is the Bayesian statistical model [36]. The acquisition function is utilized to determine the optimal sampling point, where the optimal solution is most likely to exist, or the area that has not yet been sampled. As a prior over function, the Bayesian statistical model can estimate the hypothesis posterior distribution of the function to be optimized using the prior information and observed results. To maximize the expected benefit of the new measurement points, compared with the historical optimal value, the Excepted Improvement is adopted as the acquisition function. Owing to the merits of flexibility and tractability, the Gaussian process is adopted as an a priori function of the algorithm.

**Figure 4:**
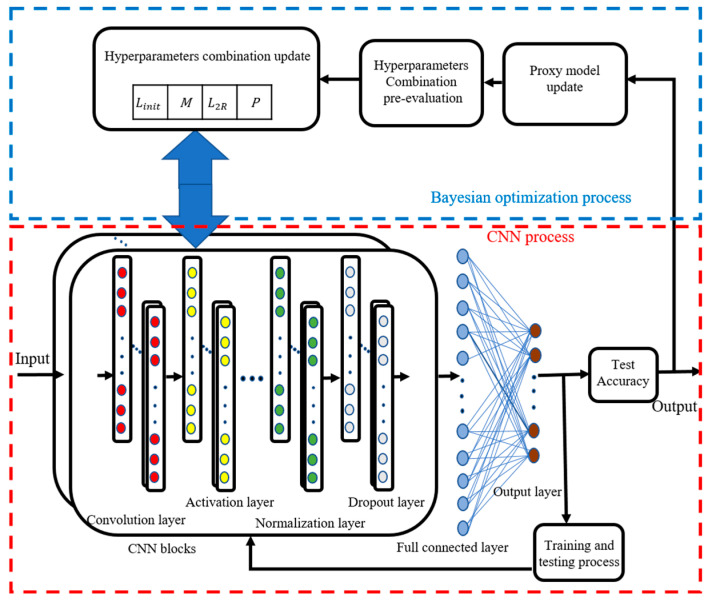
The process of hyperparameters tuning with Bayesian optimization.

The process of hyperparameter tuning using the Bayesian optimization algorithm is graphically shown in Figure 4. The whole process can be divided into two parts: the CNN process, and the Bayesian optimization process. The blue box in Figure 4 is the Bayesian optimization process, which mainly completes the initialization selection of the hyperparameter combination, and then updates it according to the previously gathered results. The red box in Figure 4 is the CNN process, which mainly completes the training and testing of deep network parameters. When reaching the termination conditions, the CNN process passes the test accuracy of the network to the Bayesian optimization process. The hyperparameters that are chosen for optimization include the Momentum (*M*), the InitialLearnRate (*L_init_*), the dropoutProb (*P*), and the L2Regularization (*L*_2*R*_). During the execution of the optimization, the two processes interact with each other until reaching the termination condition of the optimization, and then the best test accuracy, and its corresponding hyperparameters, are output as the result of the whole optimization.

### 3.5. Multiscale Kernel-Based Residual CNN Architecture

**Figure 5:**
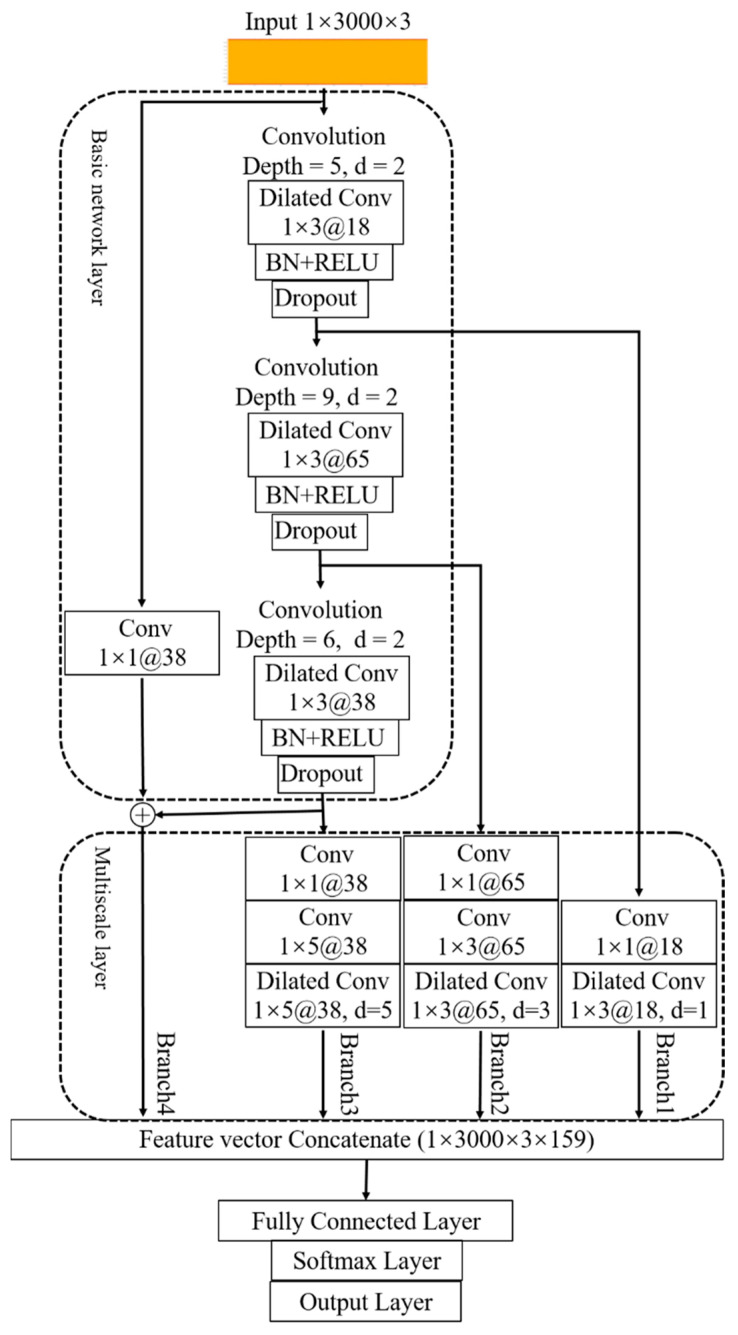
The architecture of multiscale kernel-based residual CNN. The architecture consists of four branches with kernel size and dilated factor of 1 × 3, *d* = 1, 1 × 3, *d* = 3, 1 × 5, *d* = 5, and 1 × 3, *d* = 2. The residual learning is applied in the basic network layer to prevent the degradation problem.

The structure of the multiscale kernel-based residual CNN architecture is shown in Figure 5, which is inspired by the multiscale feature extraction in image processing and speech recognition. As the figure shows, the structure of the algorithm can be divided into three parts, namely, the input part, the feature extraction part, and the output part.

In this architecture, time-series three-phase current segments are adopted as the input signal. Each current signal has a fixed size of 1 × 3000 × 3. The feature extraction part consists of two structures: one is the basic network layer, and the other is the multiscale layer. Since the dilated CNN has the advantage in feature extraction and enlarging receptive field, it was chosen for the construction of feature extraction. The basic network layer, which consists of three dilated CNN layers, is mainly responsible for the extraction of basic features. The three dilated CNN layers are used to extract the shallow, medium, and high levels of features with a layer depth of 5, 9, and 6, respectively, and a kernel size of 1 × 3. The multiscale layer, which consists of four branches, each of which represents a different feature scale, is used to extract the extra multiscale features. In each branch, the conventional convolutions are added before the dilated convolution as they can extract the information of the dilated parts, which enhances the continuity of the information. Branch 1 is used to extract the shallow level of features with a kernel size of 1 × 3 and dilated factor *d* = 1. Branch 2 is used to extract the medium-level features by a conventional convolution layer with a kernel size of 1 × 3, which can then be extracted by a dilated convolution layer with a kernel size of 1 × 3 and dilated factor *d* = 3. Branch 3 is used to extract the high-level features by a conventional convolution layer with a kernel size of 1 × 5, which can then be extracted by a dilated convolution layer with a kernel size of 1 × 5 and dilated factor *d* = 5. Branch 4 represents the residual learning result of the basic network layer features.

The features of the four branches are fused by the feature vector concatenate layer, and then pass the features of different scales to the output of the whole architecture, which consists of a fully connected layer, softmax layer, and output layer.

**Figure 6:**
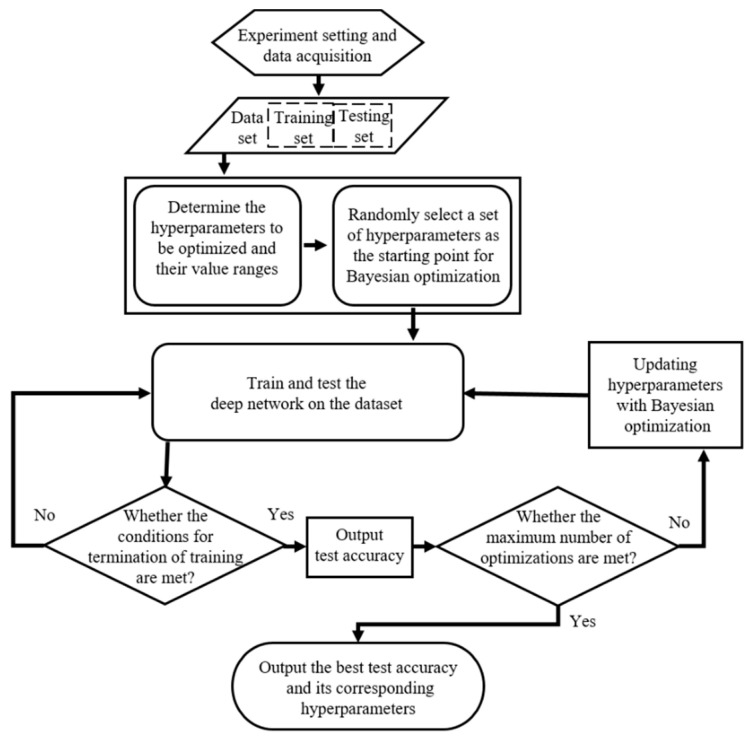
The flowchart of the multiscale kernel-based residual CNN algorithm.

The flowchart of the multiscale kernel-based residual CNN algorithm is shown in Figure 6. There are four procedures in the process:

(1) Data set preparing. The three-phase current signal is collected in experiments on the motor with an ITSC fault using the data collection equipment. Then, the acquired current signals are processed into segments of equal length and labeled with corresponding markers. After that, all the segments are split into two disjointed sets, namely, the training set and the testing set.
(2) Algorithm initialization. The value range of the hyperparameters that are optimized are determined. Then, a set of hyperparameters is randomly selected as the starting point for Bayesian optimization.
(3) Implementation of the proposed algorithm. The given starting point of hyperparameters training and testing of the proposed deep network is used, and then the hyperparameters are updated according to the Bayesian optimization algorithm. The procedure is repeated until the termination condition is met.
(4) Output results. When the maximum number of the optimization is reached, the best test accuracy and its corresponding hyperparameters of the proposed deep network are selected as the result and output.

## 4. Experiment and Data Description

**Figure 7:**
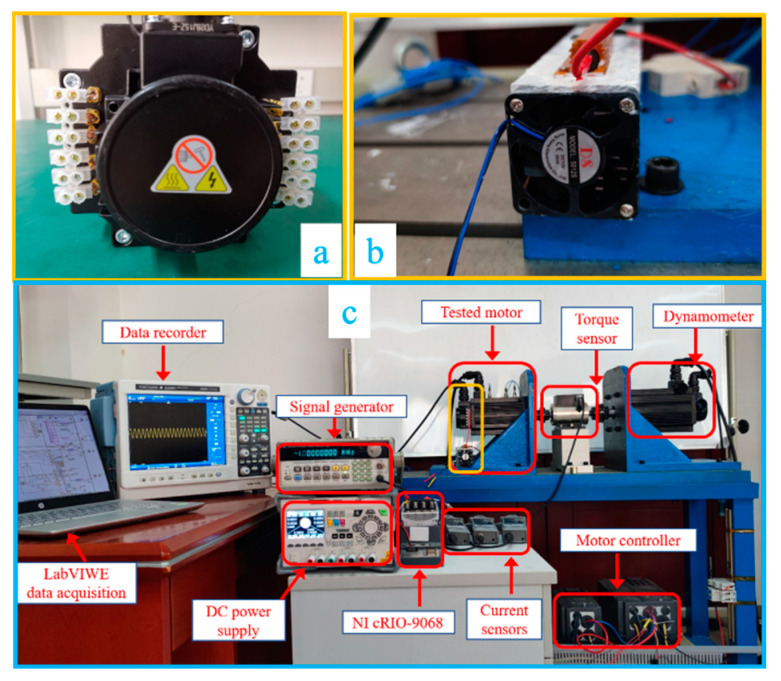
Experimental setup: (**a**) is the tested motor and its shorted points of the winding, (**b**) is the fault resistance and its heat sink, and (**c**) shows the components of the entire experiment setup.

To demonstrate the effectiveness of the proposed algorithm, an experiment on a PMSM with an ITSC fault was conducted. As shown in Figure 7, the experimental setup consisted of a dynamometer, the tested motor, a torque sensor, a data recorder, etc. The yellow box in Figure 7c was the severity setting unit of the tested motor, which contained both many shorted points of the winding (as shown in Figure 7a) and the fault resistance with its heat sink (as shown in Figure 7b). The experimental data (three-phase current of the stator) was collected with a sampling rate of 1 MHz by the DL850EA oscilloscope recorder. The tested motor was driven by a VFD037C23A inverter at a switching frequency of 15 kHz. FOC was used for motor control, where the test motor ran under constant load and the speed was determined by the dynamometer. The tested motor was wye-connected with a concentrated winding of 108 turns per phase. The main specifications of the tested motor are shown in Table 1.

**Figure 8:**
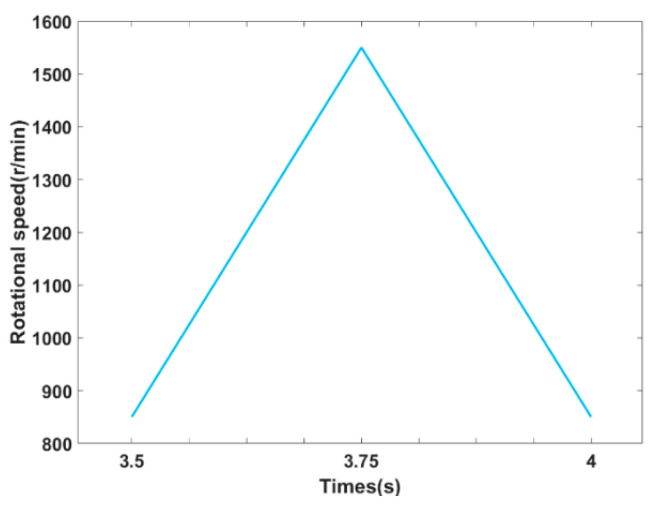
The schematic of dynamic rotational speed.

The experiment of the tested motor was conducted under various operating conditions. The operating conditions were combinations of two-load torques and five rotational speeds, as shown in Table 2. The two-load torques were both constant, while among five speeds, four were constant and one was dynamic. There were 10 operating conditions, while every operating condition was a combination of one torque and a speed. As shown in Figure 8, the dynamic rotational speed ranged from 850 to 1550 r/min, which was controlled automatically. The motor was tested in health and fault states.

**Figure 9:**
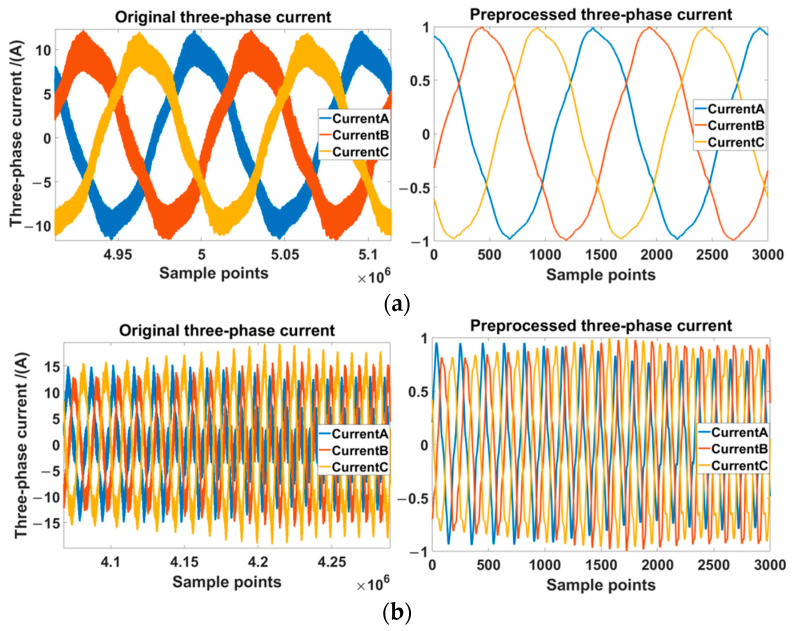
The comparison of the collected three-phase current signals before and after preprocessing. The left side of the figure is the original signal and the right side of the figure is the preprocessed signal. (**a**) The signal was collected in the healthy state of the motor at a constant speed of 150 rpm and a load torque of 3.5 N·m. (**b**) The signal was collected in a faulty state of “A6R0.1” at the set dynamic speed and a load torque of 3.5 N·m.

The severity of the ITSC fault in the tested motor could be set according to (8). In our experiment, there was 1 health state and 16 fault states, as shown in Table 3. The ITSC faults were set in phase A and the fault resistance was connected between two shorted points. There were four fault resistances and four shorted turn ratios. Each label was a combination of fault resistance and shorted turn ratio. The labels represented the state of the tested motor. “HL” represented the healthy state of the motor. “R5”, “R1”, “R0.5”, and “R0.1” represented the fault resistances of 5 Ω, 1 Ω, 0.5 Ω, and 0.1 Ω correspondingly. “A2”, “A4”, “A5”, and “A6” represented the shorted turn ratios of 4.6%, 8.3%, 10.2%, and 13.8% correspondingly. The 17 labels were sorted in ascending order according to the result calculated by (7). To prevent low-frequency aliasing interference, the acquired raw current signals were filtered by a zero-phase filter, and, then, the sampling frequency was down-sampled to 15 kHz, which was consistent with the switching frequency of the inverter. The current signals were normalized and cut into segments of the same length. The comparison of the collected three-phase current signal before and after preprocessing is shown in Figure 9. There were 20,400 segments of the acquired data, and each label was 1200. The segments in each label were randomly divided into two disjoint sets: one was the training set with 840 segments, and the other was the testing set with 360 segments.

## 5. Results and Comparisons

The proposed multiscale kernel-based residual CNN algorithm was utilized to accomplish the procedures of training, testing, and optimization offline using the current signal data sets. The optimized hyperparameters included InitialLearnRate (*L_init_*), Momentum (*M*), L2Regularization (*L*_2*R*_), and dropoutProb (*P*). The corresponding search intervals, data types, and best results of each hyperparameter are shown in Table 4. The item “Transform” indicated whether the hyperparameter was optimized on a logarithmic scale.

**Figure 10:**
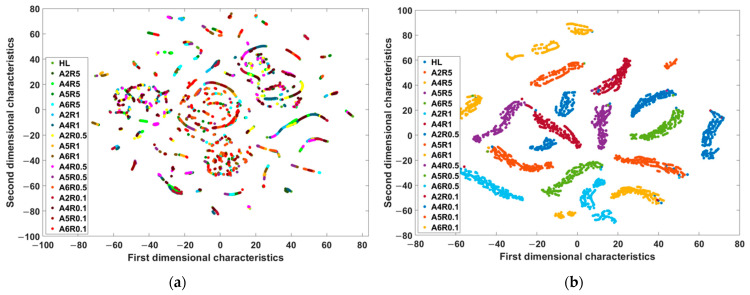
Visualizations of high-dimensional characteristic maps at different layers in the proposed algorithm. (**a**) Input characteristic map and (**b**) output of the full-connected layer characteristic map. Each point stands for a sample segment and the different colors represent corresponding severity samples.

According to the experience during the tuning process, the optimization number was set to 80, and the training epoch number was set to 90 in every single optimization. The best result of each hyperparameter is shown in Table 4. To visualize the features learned by the trained network, a data dimensionality reduction visualization algorithm, called the t-distribution stochastic neighbor-embedding algorithm (T-SNE), was used to demonstrate the learned features. The features of the input layer and the output layer were compared in a two-dimensional (2-D) form, which not only simplified the comparison but also visualized the features, as shown in Figure 10. It can be seen that there were 17 colors in both feature maps; each color corresponded to a label of the ITSC fault, which represented the fault severity. Figure 10a is the feature map of the input layer, from which we can see that the features of different severity states were heavily overlapped. It was very hard to distinguish the labels from each other in this situation. Then, as shown in Figure 10b, the features of different severity states could be easily separated from each other after the feature extraction training of the network, which exhibited the effect of the proposed algorithm.

To assess the performance of the proposed algorithm, four indicators, namely, the recall ratio (*r*), the precision ratio (*p*), the overall accuracy (acc), and the *F*1 score, were introduced to do the evaluation [37]. In massive data, the indicator of recall ratio and precision ratio are mutually constrained. The indicator of the *F*1 score simultaneously considers the effect of the recall ratio and precision ratio, which can better demonstrate the performance of the proposed algorithm [38]. The definitions of the evaluation indicators were expressed as (14) shown:

(14) $$\begin{array}{l} p=\frac{TP}{TP+FP}\\ r=\frac{TP}{TP+FN}\\ \mathrm{acc}=\frac{TP+TN}{TP+TN+FP+FN}\\ F1=\frac{2p\times r}{p+r} \end{array}$$

where *TP* represented true positive, which stood for the positive samples that were rightly classified as positive. *FP* represented false positive, which stood for the negative samples that were wrongly classified as positive. *FN* represented false negative, which stood for the positive samples that were wrongly classified as negative. *TN* represented true negative, which stood for the negative samples that were rightly classified as negative. Moreover, the positive samples stood for the samples of the current label being diagnosed, while the negative samples stood for the samples that were not part of the current label.

**Figure 11:**
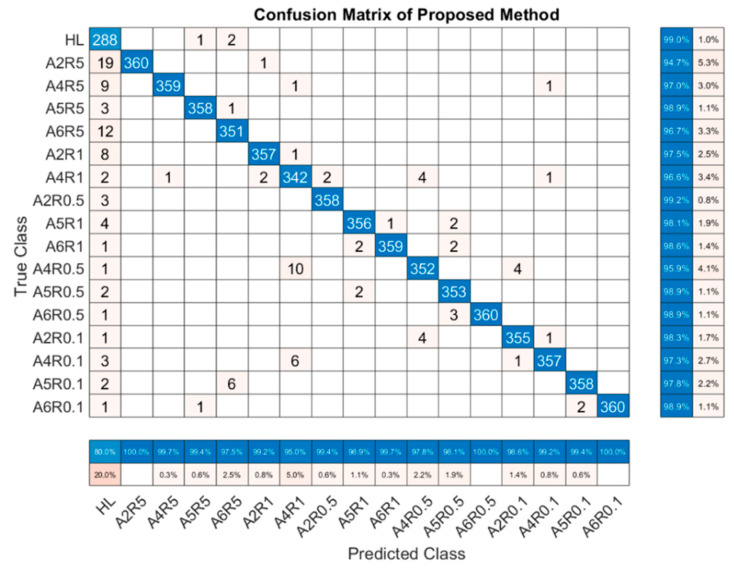
The confusion matrix of the best result in the proposed algorithm.

The overall test accuracy of the proposed algorithm, based on the best result of optimization hyperparameters, was 98.0%. To give a more detailed illustration, the confusion matrix of the beat overall test accuracy is shown in Figure 11. The labels of the confusion matrix were ranked in ascending sequence, based on the severity results, which were calculated by (8). The leftmost labels of the matrix were the true classes, which represented the true labels of the number in each row. The bottom labels of the matrix were the predicted classes, which were classified by the proposed algorithm and represented the predicted labels of the number in each column. The bottom of the confusion matrix shows the precision ratio for each label, while the recall ratio for each label is located at the rightmost of the matrix. The numbers in the blue diagonal box indicate the true positive of each label. The numbers in each column, except those on the diagonal, were false positive, while the numbers in each row, except those on the diagonal, were false negative. The precision ratio for each label was calculated as the ratio of the number on the diagonal to the sum of the rest numbers in the column. Similarly, the recall ratio for each label was calculated as the ratio of the number on the diagonal to the sum of the rest numbers in the row.

To demonstrate the superiority of the proposed multiscale architecture in handing the severity estimate of the ITSC fault, the performance of the proposed algorithm was compared with five other deep learning methods: conventional CNN, dilated CNN (d-CNN), dilated residual CNN (d-ResCNN), Long-Short Term Memory (LSTM), and Bi-directional Long-Short Term Memory (Bi-LSTM). In particular, only the methods concerned with RNN and CNN were involved in the comparison, because deep networks have an unmatched advantage over traditional machine learning methods in dealing with classification problems, while the chosen methods perform much better than other deep learning methods in dealing with time-series problems [14]. All the compared methods were optimized by Bayesian optimization, which could ensure the objectivity of the comparison, eliminating the influence of perceived subjective factors. The iteration number of Bayesian optimization was set to 80, and the result with the highest overall test accuracy was elected for comparison after the optimization was finished in each method. All the methods were trained and tested in the same dataset, while the number was set to 90 for each optimization of the training epochs. The structure and training parameters of each algorithm after Bayesian optimization are shown in Table 5.

The highest overall test accuracy of each method, and the F1 score of each label in each method, are shown in Table 6. The overall test accuracy of each method was 58.9%, 74.1%, 74.1%, 86.9%, 95.2%, and 98.0%, respectively, as shown in Table 6. The proposed method had the best performance in both the overall test accuracy and the test accuracy in each label. The performances of LSTM and Bi-LSTM were both very poor, maybe because the training of RNN was more complicated than that of CNN, requiring a longer training time or more training epoch numbers. To prove this, we increased the training epoch number to 200. After the training, the two RNN algorithms had not yet fully converged, and the Bi-LSTM performed better than the LSTM, while the overall test accuracy was less than 90%. Of course, this result did not necessarily indicate that the RNN performed worse than the CNN, but it did indicate that the RNN was much worse than CNN in terms of convergence speed. It can also be seen from Table 6 that the implementation of dilated convolution, residual learning architecture, and multiscale architecture could help enhance the performance of the conventional CNN, as the overall test accuracies of these algorithms performed better than that of the conventional CNN. In addition, the effect of dilated convolution was more pronounced than that of residual learning architecture in improving the overall test accuracy to some extent, while the residual learning architecture and multiscale architecture could further enhance the performance when the performance of the dilated CNN reached its limit. When simultaneously analyzing Figure 11 and Table 6, it could be concluded that in every method existed the same severe “false alarm rate” difficulty, which misclassified the healthy labels into faulty ones. A more serious problem was the “concealed alarm rate”, which misclassified some faulty labels into healthy ones, which might lead to catastrophic consequences. The “concealed alarm rate” was obvious in labels with slight severities as the difference of the fault features between slight severities and healthy was very little, which increased the difficulty of the classification. As the severity of the fault increased, the distinction between different fault features in each label became more apparent, making classification easier and reducing the “concealed alarm rate” significantly. Hence, in most cases, the test accuracy for each label under every algorithm increased along with the aggravation of the fault severity. In addition, it could be concluded from the ranking of the labels that the fault resistance had more effect than that of the shorted turn ratio in the early stage of the fault.

While the “false alarm rate” and the “concealed alarm rate” problems expose the fault diagnosis of an ITSC fault to some risk of misclassification, in practice these problems can be solved by continuous sampling, as the adopted dataset consists of many segments, which are portions of the data taken during a sampling period. When doing the fault diagnosis in practice, the proposed algorithm can be implemented for sequential analysis of the acquired signals, which means the result of the fault diagnosis is decided by more than one data segment. With an overall test accuracy of over 98%, the probability of two consecutive classification errors is less than 0.1%, so the fault diagnosis accuracy can be enhanced by comprehensively analyzing the diagnosis result of consecutive multiple data segments.

**Figure 12:**
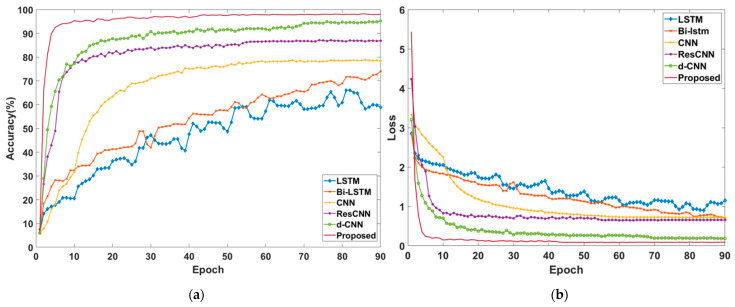
(**a**) The overall test accuracy trend in each algorithm and (**b**) the trend of the loss function in each algorithm.

To make a comprehensive analysis of the compared six algorithms, the trends of the overall test accuracy and the loss function with the growth of iterations are shown in Figure 12. The compared results in Figure 12 are the best result of each algorithm with hyperparameters tuned by the Bayesian optimization. During the complete training process, the data of each algorithm was recorded for every epoch. The trends of the overall test accuracy are shown in Figure 12a, while the trends of the loss function are shown in Figure 12b, and the corresponding trends of each algorithm in both figures were consistent. It can be seen from the figure that the overall test accuracies of LSTM and Bi-LSTM were both slowly increasing, and, up to the set maximum number of iterations, neither of the two algorithms tended to converge. The performance of Bi-LSTM was better than that of LSTM, both in the overall test accuracy and the growth rate during the whole process, and the two algorithms had potential in enhancing the performance if the iteration times could be increased. The growth rate of the conventional CNN was better than that of the two RNN algorithms, but the final overall test accuracy was no more than 80%, which was limited by its receptive field and degradation problems. The performances of ResCNN and dilated CNN were better than that of conventional CNN in terms of growth rate and the final overall test accuracy, while the dilated CNN did better than ResCNN, which proved that dilated convolution was more useful than that of residual learning connection in improving the performance of the CNN. The proposed algorithm had the best performance, with a final overall test accuracy close to 98%, which outperformed the dilated CNN algorithm by more than 2.7%. The result of the comparison demonstrated that the residual learning connection and the architecture of multiscale kernels could both help to improve the limits of a CNN architecture in feature extraction, while the architecture of multiscale kernels outperformed the residual learning connection.

In order to avoid overfitting, resulting in excessive accuracy of the algorithm, this paper also investigated the noise immunity of the algorithm to different noise levels [23]. Gaussian white noise with signal noise ratios of 15 dB, 20 dB, and 25 dB were added to the original testing dataset, respectively, to simulate the data collected from different environments [26]. The definition of the *SNR* is expressed as:

(15) $$SNR(dB)=10\log_{10}(\frac{P_{signal}}{P_{noise}})$$

where *P_signal_* represents the power of the current signal, and *P_noise_* represents the power of the noise signal.

**Figure 13:**
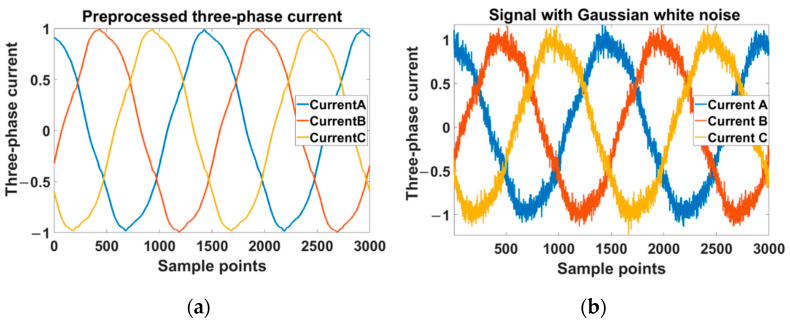
The comparison of the three-phase current signal before and after the addition of Gaussian white noise. (**a**) The signal without noise. (**b**) The signal after the addition of Gaussian white noise.

To visualize the effect of noise on the three-phase current signal, the comparison before and after the addition of Gaussian white noise with *SNR* = 20 dB is shown in Figure 13. From the figure, we can see the original signal was heavily influenced by noise and it was difficult to consider the two signals as the same one.

**Figure 14:**
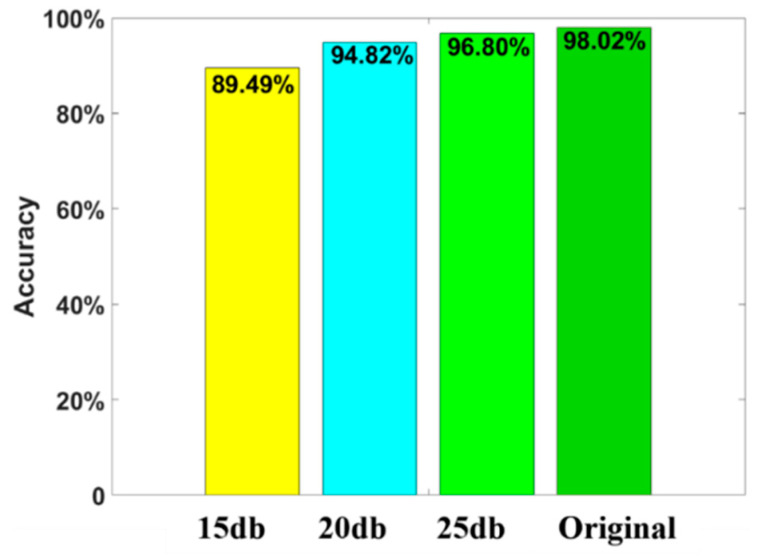
The comparison of the overall test accuracy with different levels of noise.

To test the overall test accuracy at different noise levels, the proposed algorithm was tested under the dataset with Gaussian white noise of 15 dB, 20 dB, and 25 dB, and without noise. The results are shown in Figure 14. As can be seen from the figure, the performance of the proposed algorithm decreased as the noise level increased, but the reduction was small. The results proved that the high overall test accuracy of the proposed algorithm was not due to overfitting, and the proposed algorithm had high anti-noise capability with an *SNR* greater than 15 dB.

## 6. Conclusions

In this paper, a novel multiscale kernel-based residual CNN algorithm was proposed for the ITSC fault diagnosis of a PMSM. The proposed algorithm can be implemented both in constant operating conditions and in dynamic conditions. Firstly, the dilated convolution was used to extend the receptive field of the deep network. Secondly, the residual learning connection was applied to solve the performance degradation problem with the growth of network depth. Thirdly, a multiscale kernel architecture was proposed to extract more scale features from the acquired data. Then, the Bayesian optimization algorithm was introduced to overcome the hyperparameter tuning difficulties. Furthermore, a motor fault experiment with both constant operating conditions and dynamic operating conditions was conducted by setting the fault severity of the motor to 17 levels. The proposed multiscale kernel-based residual CNN algorithm was employed to analyze the current signals that were acquired in the experiment. LSTM, Bi-LSTM, conventional CNN, ResCNN, and d-CNN were also applied to the same data set for comparison. The results illustrated the capability and impressive performance of the proposed algorithm. The results also demonstrated that the proposed algorithm is not only a fault diagnosis method, that can directly extract features from raw current signals, but can also do the severity estimate missions of the classification data under dynamic operating conditions. The multiscale kernel architecture can analyze the signals on different scales. Meanwhile, the residual connection enables the proposed algorithm to construct a deeper network, despite the degradation difficulty. Unfortunately, the proposed algorithm is only concerned with the identification accuracy of fault severity estimation under a given inverter control method, so the generalization capability of the proposed algorithm under different control methods needs to be improved. The next research will focus on extracting some features independent of the control method through preprocessing to enhance the generalization ability of the proposed algorithm to different control methods.

## Figures and Tables

**Table 1:** Specifications of the PMSM.

Parameters	Values
Pole pairs	4
Power	2.3 kW
Rated torque	15 N·m
Rated current	9.5 A
Rated speed	1500 rpm
Line-line resistance	1.1 Ω
Line-line inductance	4.45 mH
Voltage constant	114 V/1000 r/min

**Table 2:** Operating conditions of the PMSM to be tested.

Case	1	2	3	4	5
Speed (rpm)	150	450	900	1350	850–1550–850
Torque (N·m)	3.0				
	7.5				

**Table 3:** Fault data description.

Label	Fault Setting		Sample Size		
	Fault Resistance (Ω)	Shorted Turn Ratio (%)	Training	Testing	Total
HL	Inf	0	840	360	1200
A2R5	5	4.6	840	360	1200
A4R5	5	8.3	840	360	1200
A5R5	5	10.2	840	360	1200
A6R5	5	13.8	840	360	1200
A2R1	1	4.6	840	360	1200
A4R1	1	8.3	840	360	1200
A2R0.5	0.5	4.6	840	360	1200
A5R1	1	10.2	840	360	1200
A6R1	1	13.8	840	360	1200
A4R0.5	0.5	8.3	840	360	1200
A5R0.5	0.5	10.2	840	360	1200
A6R0.5	0.5	13.8	840	360	1200
A2R0.1	0.1	4.6	840	360	1200
A4R0.1	0.1	8.3	840	360	1200
A5R0.1	0.1	10.2	840	360	1200
A6R0.1	0.1	13.8	840	360	1200

**Table 4:** Hyperparameters to be optimized.

Hyperparameters	Search Intervals	Data Types	Transform	Best Result
*L_init_*	[1 × 10^−5^ 1]	real	log	3.6838 × 10^−4^
*M*	[0.5 1]	real	log	0.9031
*L*_2*R*_	[1 × 10^−10^ 1 × 10^−2^]	real	log	8.4497 × 10^−10^
*P*	[1 × 10^−5^ 1]	real	log	0.1868

**Table 5:** The structure and training parameters of the compared algorithms.

Items	LSTM	Bi-LSTM	CNN	ResCNN	d-CNN	Proposed
Layer1	Filter = 66 Hidden units = 72	Filter = 128 Hidden units = 85	Filter = 3 Depth = 1	Filter = 16 Depth = 2	Filter = 4 Depth = 6	Filter = 18 Depth = 5
Layer 2	Filter = 66 Hidden units = 65	Filter = 128 Hidden units = 100	Filter = 148 Depth = 7	Filter = 120 Depth = 6	Filter = 40 Depth = 11	Filter = 65 Depth = 9
Layer 3	Filter = 66 Hidden units = 87	Filter = 128 Hidden units = 82	Filter = 12 Depth = 2	Filter = 24 Depth = 6	Filter = 37 Depth = 4	Filter = 38 Depth = 6
*L_init_*	0.0040	9.7603 × 10^−4^	7.016 × 10^−6^	6.531 × 10^−4^	3.2541 × 10^−4^	3.6838 × 10^−4^
*P*	0.0992	0.0056	0.0312	0.0010	1.0009 × 10^−5^	0.1868
*L*_2*R*_	__	__	0.0095	4.220 × 10^−4^	0.0083	8.4497 × 10^−10^
*M*	__	__	0.9166	0.0789	0.8199	0.9031

**Table 6:** Comparison with other algorithms.

Label	LSTM (%)	Bi-LSTM (%)	CNN (%)	ResCNN (%)	d-CNN (%)	Proposed (%)
acc	58.9	74.1	78.5	86.9	95.2	98.0
HL	36.51	47.21	28.99	65.35	78.9	88.49
A2R5	50.53	61.51	61.50	84.90	92.76	97.28
A4R5	56.89	61.91	68.91	82.39	94.22	98.33
A5R5	53.88	88.03	83.38	93.43	96.40	98.64
A6R5	50.61	79.60	77.34	90.92	93.10	97.10
A2R1	55.43	67.99	64.61	82.66	95.11	98.34
A4R1	56.68	55.05	56.06	75.64	91.43	95.79
A2R0.5	58.64	68.41	68.91	86.35	95.60	99.30
A5R1	48.08	80.30	78.62	90.72	93.60	98.50
A6R1	45.22	84.41	80.72	89.92	97.65	99.15
A4R0.5	62.54	66.64	63.25	82.05	90.80	96.84
A5R0.5	51.27	79.43	77.19	89.95	95.59	98.50
A6R0.5	54.57	83.72	77.44	87.96	97.13	99.45
A2R0.1	70.75	75.31	66.02	89.20	96.05	99.45
A4R0.1	77.28	78.43	64.73	95.43	97.80	98.24
A5R0.1	81.37	87.80	85.69	93.94	98.45	98.59
A6R0.1	87.80	86.77	88.97	96.15	98.65	99.45
